# Pesticide Safety Awareness among Rural Farmers in Dadinkowa, Gombe State, Nigeria

**DOI:** 10.3390/ijerph192113728

**Published:** 2022-10-22

**Authors:** Haruna Musa Moda, Daniel Mensah Anang, Newton Moses, Felix Mandoli Manjo, Victoria Ibukun Joshua, Nwadike Christopher, Paulina Doka, Mela Danjin

**Affiliations:** 1 Department of Health Professions, Faculty of Health and Education, Manchester Metropolitan University, Manchester M15 6BG, UK; 2 Department of Horticultural Technology, Federal College of Horticulture, Dadin-Kowa 761121, Gombe State, Nigeria; 3 Department of Public Health, College of Nursing and Midwifery, Jos 760251, Gombe State, Nigeria; 4 Department of Agricultural Technology, Federal College of Forestry, Jos 930253, Gombe State, Nigeria

**Keywords:** low- and middle-income countries, pesticide exposure, safety awareness, public health, pesticide poisoning, rural farmers

## Abstract

Introduction: Because of the longer growing season and warmer climate, weeds and insect pests spread are on the rise, thereby increasing the demand for pesticide use and consequently harmful emissions that further exacerbate climate change. Unsafe occupational exposure to pesticide residue is associated with a lack of product knowledge and safety awareness among farmers in low- and middle-income countries (LMICs). Materials and Methods: A cross-sectional descriptive study design was adopted for this study in which a face-to-face administered questionnaire was used to collect data from 285 respondents who were selected using convenient snowball sampling technique. Knowledge, awareness, and practices related to pesticide storage, handling, application, and containers disposal among the farmers were measured. Categorical variables were analysed and presented using descriptive statistics in the form of frequency count and percentage, while numeric items were summarized using mean and standard deviation. Results: Dichlorvos and Perfekthion 2.5 EC listed in the WHO Group I pesticide classification were among the most frequently used pesticides. Symptoms of pesticide intoxication reported include headaches (56.1%), dizziness (56.5%), skin irritation (53.3%), and fatigue (45.6%), respectively. Farmers’ behaviour during pesticide application include blowing clogged nozzle with mouth (42.7%), talking while spraying (59.8%), and mixing pesticide with bare hands (31.1%). Furthermore, 38.5% of them use pesticide containers for other domestic purposes. Conclusions: Socioeconomic factors, i.e., educational level, age, and years of farm practice, influenced farmers safety behaviour. Based on these findings, an approach that will help strengthen capacity building programmes and the enhancement of knowledge-based initiatives around the adoption of non-synthetic pest-control methods should be encouraged.

## 1. Introduction

Pesticide use is synonymous with farming communities, especially in low- and middle-income countries (LMICs), with the wide spread of acute pesticide poisoning affecting farmers, farmworkers, and their families due to non-existent or inadequate safety and health standards [1,2,3]. Because of the high dependency on pesticide use among farmers to protect crops against pests such as desert locust in parts of Africa and to maximise crop yield, the exposure route to pesticide among farming communities is prevalent and can either be in non-occupational settings via residue in drinking water and food, which might be at low levels [4,5], and occupational, i.e., through inhalation or oral and dermal contact, where the farming community is exposed to these pesticides on a regular basis with resultant ill health symptoms, which might be immediate or after long periods of exposure [2,6]. Previous studies have established the relationship between pesticide exposure and resultant adverse effects that involve biomolecular alterations leading to the development of related diseases, especially among vulnerable groups that include children, pregnant women, individuals with compromised immune systems, etc. [7,8,9].

Despite the high burden related to acute pesticide poisoning reported at both global and regional levels, due to limited access to information, training, and appropriate equipment to safely apply pesticide on farms, several famers and farmworkers, especially in LMICs, lack safety and risk awareness related to pesticide storage, use and disposal of empty containers, non-use or inappropriate use of PPE, and the lack of infrastructure and resources by governments to regulate and monitor pesticide use [2,8,10,11]. These alongside other factors contribute to the high morbidity and mortality rates associated with pesticide exposure among the farming communities.

Although the impact associated with pesticide use in developing countries might be underestimated, the high use of highly hazardous pesticide (HPPs) presents a significant clinical impact among farming communities, including respiratory, integumentary, cardiovascular, gastrointestinal, and neurological effects [11,12,13]. A report on pesticide and HHPs in Nigeria revealed that at the end of the 2018 financial year, 147,446 tons of pesticides were imported into the country, and 584 tons of the imported stocks were in the HHPs classification group [14]. The north-eastern region of Nigeria plays a crucial role towards the production of both farm and animal produce that supports the federal government’s food security agenda. The present government’s drive for agricultural practices in the country comes with a high use of pesticides, which mostly are products imported into the country for high crop yield. Earlier [13] illustrated the safety challenges posed by pesticide use within the agricultural sectors of developing countries to include the underestimation of pesticide harm to human health, misunderstanding of safety guideline among the farming communities, infiltration of counterfeit pesticide into the local market, and lack of regulations, which paves the way for profit over human safety. As such, the need to understand the extent to which pesticide can pose risks to farmers’ health will require the government’s and relevant stakeholders’ effort to develop an effective monitoring and testing regime to ensure their safe usage and handling are considered during application and storage. The present study measured rural farmers’ safety knowledge, awareness, and practices as they relate to pesticide application and storage.

## 2. Materials and Methods

### 2.1. Study Area, Population, and Sampling

The study was conducted between March–June 2021, in Dadinkowa, located 35 km away from Gombe, the state capital in the north-eastern part of Nigeria. The study area is in the Yamaltu-Deba local government area, with a projected population of 348,019 inhabitants, sharing boundary with the Akko local government area to the south-west, Deba to the east, and Kwami to the north. The area has an altitude of about 370 m above sea level and houses the multipurpose Dadinkowa dam, which has a surface area of 3000 square km with 1.7 million m^3^ constructed holding capacity. The aim of the dam construction includes water provision for domestic use, irrigation, fish farming, and electricity generation within the area, and its surrounding environment provides employment to 71% of the inhabitants. Two distinct seasons, namely wet (April–October) and dry season (November–March), characterise the region, with an average rainfall of 850 mm and a mean annual temperature of 32 °C. Different horticultural crops produced in the regions include tomatoes, vegetables, fruit, cotton, cereal, etc., as major crops cultivated in the area for local use of the community and export to various region within Nigeria and to the neighbouring countries of Chad and Cameroon.

Based on frequently reported pesticide intoxication symptoms among the target population, a cross-sectional study was adopted to help make inference regarding the possible relationship between health symptoms associated with pesticide exposure and farmers’ attitude to safety and health. Based on this, a structured questionnaire was adopted to collect required information among 285 farmers located within the study area.

Inclusion criteria included farmers or farm workers involved with pesticide-application-related tasks that include pesticide mixers, pesticide sprayers, and responsible purchase and storage of pesticides and falling within the age group of above 18 years. Participation was voluntary, and recruitment was achieved with the assistance of local farmers’ association and farm extension officers that helped with the sensitization exercise among the farming community. A convenient snowball sampling technique was adopted to select the participants.

Questions asked include personal data, types of pesticide used, experience of pesticide intoxication, hygiene routine while handling pesticide, attitude practice and knowledge around pesticide safety, and preferred pesticide information source among others. Trained interviewers fluent in the local dialects (Hausa and Tera) commonly spoken among the farming community administered the face-to-face questionnaire. The technique helped to reassure participants and provide needed information to help respondents decide in taking part or otherwise. This also takes into consideration that most farmers are likely to have limited formal education and may not be familiar with the terminologies used in the questionnaire. The Federal College of Forestry ethics committee, Jos (FCFJ/MMU/001/02/2021), granted ethics approval on 9 February 2021.

### 2.2. Data Analysis

The data analysis was conducted using IBM SPSS version 27 IBM United Kingdom Limited, Cheshire, UK. Categorical variables were analysed and presented using descriptive statistics (frequency counts and percentages), while numeric items were summarized using mean and standard deviation. Reliability for sets of latent variables within each domain used in the survey instrument was assessed using Cronbach’s α test. The outcome of the analysis revealed the sets of items as closely related, with a good-to-excellent alpha score array of 0.788–0.940 across the four domains measured (Table 1).

## 3. Results

Sociodemographic variables considered in the survey are presented in Table 2. The majority of participants were within the active population age band of >20 to 50 years of age. Based on the age grouping used, those that were identified within the age bracket of 20–30 of years accounted for 29.8% of the study population. Only 12.4% of the participants said they had no formal education. A slightly higher number (63.2%) said they practice both wet- and dry-season farming. The number of years working with pesticide on farms ranged from 1 to more than 20 years among the surveyed population, and 26.4% affirmed pesticide use of more than three times per farming cycle (Table 2).

To have a clearer picture of pesticide types commonly used among the surveyed group, a list of pesticides commonly sold among vendors within the community was compiled. From their response, products classified as highly hazardous pesticide (HPPs) were among the common pesticides used on farms among the group. The organophosphate pesticide group was found to be intensively used among the farmers, with 49.8% of the participants admitting having used glyphosate at some point, and Malathion (41.8%) followed closely. Paraquat, a Group II WHO-classified product, is the most highly utilised (53%) pesticide among the participants and is closely followed by Lambda-cyhalothrin, which 52.3% of the respondent said to have applied on the farm at some point. Dichlorvos, a WHO Group I pesticide, was found to have been used by 29.8% of the farmers (Table 3).

Pesticide poisoning was prevalent among the sampled population, where each affirmed to have experienced one form of pesticide intoxication symptoms after application of the product on the farm. The most prevalent symptom reported among the group after pesticide application was dizziness (56.5%), followed by headaches (56.1%). Other symptoms reported include skin irritation (53.3%), itchy eyes (40.7%), excessive salivation (44.2%), and mucus build up in airways (22.8%) (Table 4).

Table 5 presents the assessment of farmers’ pesticide safety knowledge and their varied degree of response across the questions asked. From the result, 89.8% acknowledged that pesticide does present human health effects if not properly handled. Only 57.9% said that they either read the product label or safety data sheet prior to mixing and application of the pesticide on their farm. In addition, 54.7% of the respondents affirmed to knowing how best to dispose of either expired product or residue after application on the farm. In response to the question about knowledge on farmers’ exposure route of pesticide, 38.6% said they are aware of other secondary routes, i.e., drinking water, food contamination, etc., while the majority (60%) considered oral ingestion as the main possible route of exposure during pesticide application. The question about the use of the mouth during pesticide application to blow out a clogged nozzle revealed that 39.3% of the respondents engaged in this form of practice at some point. Regarding the use of bare hands in mixing pesticide during preparation and its application on the farm, 31.1% admitted to having engaged in this form of practice. It was further revealed that a high number of the sampled population (38.5%) use empty pesticide containers for either farm or domestic use (Table 5).

Spearman’s rho correlation coefficient was used to assess the relationship between sets variables regarding participants’ awareness and attitude towards pesticide safety (Table 6). The outcome from the result showed no significant correlation between participants’ pesticide safety knowledge and years working with pesticide on the farm (r_s_ = 0.11, *p* = 0.063). In addition, from the computed data, the result indicates that there was a significant association between pesticide handling and storage and gender among the farmers (r_s_ = 0.238, *p* < 0.01). However, a negative correlation was also established between education and pesticide application and storage among the farmers (r_s_ = −0.276, *p* < 0.01). The relationship between farmers’ safety knowledge and attitude towards pesticide safety also indicates a significant negative association (r_s_ = −0.195, *p* < 0.01).

## 4. Discussion

As a result of frequent use of pesticides for agricultural practices and the impact of climate change, which has further increased farm pest and disease resistance, the present study measured pesticide safety awareness and attitude during application and storage among farmers involved in wet- and dry-season farming in Dadinkowa and its surrounding environment. To date, there are limited studies on pesticide safety awareness among rural farmers in northern Nigeria, and this study further demonstrates the need for stakeholders’ intervention regarding safe pesticide application among this group of farmers. To demonstrate the relationship between improved personal hygiene and pesticide exposure effect, Keifer [15] affirmed that around 80% probability of poisoning avoidance is likely with combined safety and hygiene precaution during pesticide application. Such uptake among the farmers considered in the present study was limited, and those that said they do not make use of PPE blamed extreme weather condition where temperature reaches ~40 °C at its peak; this poses a major factor restricting the farmers from using any form of PPE. A similar claim was also observed among farm workers in Kuwait [16].

Several short- and medium-term post-pesticide exposure symptoms that include headache (56.1%), skin irritation (53.3%), mental confusion (21.8%), increased breathing rate (24.6%), diarrhoea (33.3%), muscular twitching (29.5%), extra phlegm in airways (22.8%), etc., were reported among the sampled group. The symptoms described among the group correspond with limited application of personal safety precautions during pesticide preparation, application, and storage, which present a source of pesticide exposure. It was found that around one-quarter of the respondents affirmed to have either used their hands in stirring pesticide, blown a clogged nozzle with their mouth (42.7%), or used a pesticide container for either farm or domestic use. This form of negative attitude was reported in other studies [16,17] In addition, other poor personal hygiene and safety practices reported include limited use of coveralls and respirators (PPE); smoking during application and eating kola nuts among the farmers were other sources of pesticide exposure, coupled with the poor practice of not referring to manufacturer instructions either on the product label or safety data sheet (SDS), further demonstrating the rationale behind the high rate of individual symptoms reported among the group. These symptoms of pesticide intoxication as reported among these farmers correlate with their behaviour during pesticide application, and a high percentage of the farmers reported having experienced various forms of symptoms during or after application. Hence, there is a need for the government and relevant stakeholders to strengthen pesticide safety awareness training to help minimise associated health and environmental risks.

From the results of the study, high use of pesticides among the farmers to control farm pests was found; however, the required level of safety practices during the handling of these products was found to not be in practice by some of the respondents. Pesticide groups that include organophosphate, triazine derivative, organochlorine, and dithiocarbamate were among the most frequent pesticides used among the farmers. As such, there is ground to express occupational risk concerns related to pesticide application and storage. Despite the indication of awareness of both human health effects (91.1%) and environmental effects (79.6%) associated with pesticide exposure by the majority of farmers, reading of the safety data sheet (SDS) or product label before application was low, as only 57.9% said they engage with the information source. A high proportion of the participants said they consider the vendor’s pep talk during purchase as a sufficient guide on how best to apply and store the product. Similar outcomes were reported among farming communities in Ethiopia [18] and vegetable farmers in Ghana [19]. Similarly, regarding having the needed knowledge to enable the safe disposal of expired pesticides and or residue after use, only 54.9% of the farmers affirmed having awareness of such best practices. This further demonstrates the limited access to safety training regarding the handling and disposal of pesticides in general among the framers. This finding was consistent with an earlier study by [2,8,20], where knowledge regarding safe disposal of pesticide residue, containers, and expired products was low among farmers in rural irrigation villages in southwest Ethiopia. In addition, the shortfall in this further raises safety and health concerns for both the farmers and their families, as earlier studies have demonstrated correlation between miscarriages, especially during the first trimester; irregular menstrual flow; reproductive health; and infants’ death due to pesticide exposure related to farm practices [21,22,23,24]. Considering the role played by pesticide vendors and the high assurance around safe application methods that are relied upon by the farmers and originating from the pep talks they are involved with from the vendors, there is the need for relevant agencies, extension workers, and other related stakeholders to ensure pesticide vendors are engaged in periodic training to help increase their technical competence around pesticide safety knowledge and risk communication, which is expected to have a positive domino effect on farmers’ safe use of pesticide on farms [16,25,26].

The outcomes of the study show 29.8% of the farmers alluded to have used highly hazardous pesticides (HHP) at some point in addition to varied products classed as moderately hazardous (Class II) based on WHO classification [27]. Similarly, 78.3% of the farmers surveyed acknowledged to pesticide storage at home, indicating a high probability of exposure for both farmers and their family members. Earlier studies found a similar practice among cocoa farmers in southwestern Nigeria [28], rural farmers in Tanzania [25,29], and a rural irrigation farming community in Ethiopia [8,30]. Considering that 56.3% of the pesticides used among the farmers surveyed were among the WHO hazard Class II, it raises further safety and health concerns about the farmers as end users and the potential exposure to highly harmful agents, especially when they are stored in homes or transported with other media. The use of such harmful class agents corroborates earlier studies where pesticides used among farmers in Bangladesh (66%), Burkina Faso (65%), and Ethiopia (69%) were in the range of the WHO hazard classification II [8,31,32]. Regarding this, it is safe to conclude that efforts made at limiting pesticide exposure among the farmers considered in the present study have been unsatisfactory, and hence, there is a need to encourage alternative practices around farm pest control that could include the use of bio-control methods as well as control of the use of hazardous pesticides while ensuring that good agricultural practices are encouraged among the farming communities.

While the study did demonstrate the need for the enhancement of health and safety awareness among farmers in the region, there are limitations that are worth noting in the study. It cannot be said whether other underlying medical health statuses that present similar post-pesticide symptoms skewed the response of those that reported experiencing ill health conditions, which our study did not consider. Owing to the limited number of farmers considered, the result may not reflect the awareness and practices of other farmers outside the study area; as such, further study that includes large-scale participants is recommended.

## 5. Conclusions

While highly negative human and environmental effects associated with pesticide exposure were established, there was low uptake on safety label and SDS use prior to application and storage of pesticides among the farmers, demonstrating a negative correlation between safety awareness and practices about the handling of pesticides. Considering that both dry- and wet-season agricultural activities take place within Dadinkowa and its environment, due to the presence of the dam that supports all-season farming, the present study represents an initial attempt to bring to light the need for more intervention around safe pesticide application and storage practices. This can help reduce the health burden experienced in the area and move the state towards meeting its commitment to sustainable development goals.

## Figures and Tables

**Table 1:** Pesticide Knowledge, Attitude, and Practice Domains: Reliability and Means.

SN	Pesticide KAP Domain	No of Items	Cronbach’s Alpha	Likert Scale Range	Mean ± SD
1	Pesticide Knowledge	9	0.861	1–3	1.53 ± 0.6266
2	Pesticide Attitude	13	0.788	1–5	2.84 ± 0.9056
3	Pesticide Personal Protective Practices (PPP)	10	0.940	1–3	1.57 ± 0.4704
4	Pesticide Application and Storage Practices (ASP)	11	0.869	1–2	1.92 ± 0.8244

**Table 2:** Sociodemographic Characteristics of the Study Population.

Variable	Category	n	%
Age group			
	<20	35	12.3
	20–30	85	29.8
	31–40	58	20.4
	41–50	67	23.5
	51–60	26	9.1
	>60	14	4.9
Gender			
	Male	176	62.4
	Female	91	32.3
	Prefer not to say	15	5.3
Highest level of education			
	No formal education	35	12.4
	Primary school	30	10.6
	Secondary	98	34.6
	Tertiary	120	42.4
Smoking habit			
	Smoker	29	11.3
	Never smoked	191	74.6
	Quit smoking	36	14.1
Number of years working with pesticide on farm			
	1–5	3	1.1
	6–10	96	33.7
	11–15	100	35.1
	16–20	67	23.5
	>20	19	6.7
Work shift on farm			
	Full day	153	56.9
	Half day	116	43.1
Do you practice wet- and dry-season farming?			
	Yes, I practice both	180	63.2
	No, wet season only	85	29.8
	No, dry season only	20	7.0
How often do you apply pesticide per cropping season?			
	Once	28	11.2
	Twice	95	38.0
	Thrice	61	24.4
	More than three times	66	26.4
What land-tenure system do you presently hold?			
	Landowner	94	33.9
	Private	114	41.2
	Government	36	13.0
	Communal land tenure	33	11.9
What is your farm size?			
	Less than 1 hectare	106	40.6
	More than 1 hectare	155	59.4

**Table 3:** Inventory of pesticide types used during the last two farming seasons.

Pesticides Used or Applied Before (N = 285)	Percentage	* WHO Classification	Manufacturer Health Hazard Classification	Chemical Group
Malathion (Malataf) ^#^	41.8	III	H302, H317, H410	Organophosphate
Paraquat (Weedcrusher) ^#^	53.0	II	H311, H330, H315, H410	Dipyridilium derivative
Atrazine (Delzine, Atrataf, Atraforce, Xtrazine) ^#^	51.6	III	H317, H373, H410	Triazine derivative
Butachlor (Butaclear, Risene, Teer, Butaforce, Cleweed) ^#^	44.2	III	H302, H411	Chloroacetanilides
Glyphosate (Round-up, Wipeout, Clearweed, Bushfire) ^#^	49.8	II	H312, H318, H411	Organophosphorus
Basagran (Basagran) ^#^	29.5	II	H302, H319, H317, H412	Dithiocarbamate
Lambda-cyhalothrin (Karate Laraforce, Attack, Karto, Zap) ^#^	52.3	II	H304, H315+H320, H332, H371, H410	Pyrethroids
Propanil (Propacare, Propan, Rhonil, Orizo, Propaforce) ^#^	36.5	II	H302, H400, H411	Oxadiazon
Pendimethalin (Stomp, Pendilin) ^#^	28.4	II	H304, H317, H410	
Oxidiazon (Ronstar, Riceforce, Unicrown) ^#^	33.3	II	H304, H315, H336, H410	Oxadiazole
Mancozeb (Z-force, Hi-shield, Mancozeb, Mycotrin) ^#^	42.8	U	H317, H361d, H400	Dithiocarbamate
Dichlorvos (Smash, Wonder, Shooter, Nopest, DDforce, VIP) ^#^	29.8	I	H225, H301, H311, H317, H331, H370, H400	Organophosphate
Cypermethrin (Suraksha, Superthrin, Best, Cymbush, Cypercot) ^#^	43.9	II	H301, H317, H332, H335, H410	Organochlorine
2,4-D Amine (Aminoforce, Delmin-forte, 2,4-D-Amine, Select) ^#^	49.1	II	H302, H312, H332	Organochlorine
Dimethoate (Perfekthion, Cygon, Rogor, Daphene, Racelate) ^#^	41.8	III	H226, H302, H331, H317, H400, H411	Organophosphate
Thiamethoxam (Helix XTra, Cruiser)	44.2	III	H302, H400, H410	Neonicotinoid
Sevin (Tricarnam, Carbaryl, Vetox, Ravyon)	75.8	III	H302, H312, H332	Organic carbamate

^#^ Common trade name of products. * WHO classification: I, highly hazardous; II, moderately hazardous; III, slightly hazardous; U, unlikely to present acute hazard in normal use.

**Table 4:** Prevalence of Symptoms of Pesticide Intoxication among Farmers.

SN	Symptoms after Pesticide Application/Use	Percentage
1	Headache	56.1
2	Dizziness	56.5
3	Skin irritation	53.3
4	Vomiting	45.6
5	Nausea	37.5
6	Itchy eyes	40.7
7	Coughing	45.3
8	Stomach ache	42.8
9	Poor vision	29.8
10	Shortness of breath	35.1
11	Excessive sweating	36.1
12	Weakness or fatigue	45.6
13	Diarrhoea	33.3
14	Restlessness	23.5
15	Excessive salivation	44.2
16	Chemical burns on the skin	46.0
17	Mental confusion	21.8
18	Muscular twitching	29.5
19	Increased rate of breathing	24.6
20	Extra phlegm or mucus in the airways	22.8

**Table 5:** Respondents’ awareness of pesticide safety and route of exposure.

Variables	Total (n = 285)	
	n	%
Pesticide can affect human health (n = 281)	256	91.1
Pesticide can affect livestock health (n = 281)	226	79.6
Pesticide can affect the environment (n = 284)	196	69
Storage of pesticides safely (n = 282)	173	61.7
I have knowledge of how best to dispose of expired pesticide/residue (n = 284)	156	54.9
Personal protection usage (n = 284)	180	63.4
Safe application of pesticide on farm (n = 284)	178	62.7
Consult label/safety data sheet supplied (n = 285)	165	57.9
I talk while mixing or spraying (n = 246)	147	59.8
I eat kola nut/food while mixing or spraying (n = 239)	31	13
I consume water while mixing or spraying (n = 239)	31	13
I smoke while mixing or spraying (n = 242)	43	17.8
Storage of pesticide at home (n = 258)	202	78.3
Stir/scoop pesticide with hands (n = 238)	74	31.1
Spray pesticide along wind direction (n = 239)	180	75.3
Use of empty pesticide container for other purposes/use in the house/farm (n = 262)	101	38.5
If the nozzle gets blocked, I blow it with my mouth to get the clog out (n = 262)	112	39.3
Supposed exposure route of pesticide poisoning (n = 285)		
Dermal/skin	160	56.1
Oral/mouth	173	60.7
Eye	146	51.2
Inhalation	161	56.5
Secondary route (drinking water/food)	110	38.6

**Table 6:** Spearman’s rho correlations between sets of variables considered.

Items/Variables	Stat Parameter	1	2	3	4	5	6	7	8	9	10
Pesticide safety Knowledge of farmers (1)	R	1.000									
	*p*-Value	.									
Pesticide safety Attitude of farmers (2)	R	−0.195 **	1.000								
	*p*-Value	0.001	.								
Pesticide application and storage practice (3)	R	0.525 **	−0.245 **	1.000							
	*p*-Value	0.000	0.000	.							
Personal protective practice use (4)	R	0.102	0.075	−0.032	1.000						
	*p*-Value	0.099	0.226	0.614	.						
Age group (5)	R	−0.071	0.061	−0.044	0.071	1.000					
	*p*-Value	0.232	0.302	0.471	0.250	.					
Gender (6)	R	0.439 **	−0.086	0.238 **	−0.029	−0.044	1.000				
	*p*-Value	0.000	0.151	0.000	0.637	0.457	.				
Education (7)	R	−0.302 **	0.040	−0.276 **	−0.083	0.179 **	0.089	1.000			
	*p*-Value	0.000	0.502	0.000	0.180	0.003	0.138	.			
Years working with pesticide on farm (8)	R	0.110	−0.078	0.095	−0.066	0.406 **	0.125 *	0.063	1.000		
	*p*-Value	0.063	0.189	0.117	0.286	0.000	0.035	0.289	.		
Work shift on farm (9)	R	0.099	−0.112	0.165 **	−0.245 **	0.019	0.221 **	0.253 **	0.051	1.000	
	*p*-Value	0.104	0.066	0.008	0.000	0.758	0.000	0.000	0.401	.	
Wet- and dry-season farming (10)	R	0.268 **	−0.048	0.140 *	0.033	0.069	0.326 **	−0.006	0.100	0.243 **	1.000
	*p*-Value	0.000	0.420	0.021	0.599	0.244	0.000	0.922	0.092	0.000	.

**, Correlation is significant at the 0.01 level (2-tailed). *, Correlation is significant at the 0.05 level (2-tailed).

## Data Availability

The data presented in this study are available in the article.
